# Olfactory cleft proteome does not reflect olfactory performance in patients with idiopathic and postinfectious olfactory disorder: A pilot study

**DOI:** 10.1038/s41598-018-35776-8

**Published:** 2018-12-03

**Authors:** Axel Wolf, Laura Liesinger, Stefan Spoerk, Matthias Schittmayer, Doris Lang-Loidolt, Ruth Birner-Gruenberger, Peter V. Tomazic

**Affiliations:** 1 Department of Otorhinolaryngology, Medical University of Graz, Auenbruggerplatz 26, 8036 Graz, Austria; 2 Gottfried Schatz Research Center, Medical University of Graz, Stiftingtalstrasse 24, 8010 Graz, Austria; 3 Omics Center Graz, BioTechMed–Graz, Stiftingtalstrasse 24, 8010 Graz, Austria

## Abstract

Technical advances including liquid chromatography–tandem mass spectrometry and its data analysis enable detailed proteomic analysis of the nasal mucus. Alterations of the nasal mucus proteome may provoke substantial changes of the nasal physiology and have already been associated with rhinologic diseases such as allergic rhinitis. This study was conducted as a pilot study to map the olfactory cleft proteome using current techniques for proteomic analysis. Furthermore, we aimed to investigate proteomic changes as potential biomarkers in patients suffering from idiopathic and postinfectious olfactory disorders compared to healthy controls. Seven patients with idiopathic hyposmia and anosmia, seven patients with postinfectious hyposmia and anosmia and seven healthy controls were included in this study. In total, 1117 different proteins were detected in at least five patients in at least one group. Results of this study did not reveal significant differences regarding the proteomic composition of the olfactory cleft mucus between patients versus healthy controls. Among proteins involved in olfactory perception the G protein family was detected but also found unchanged between groups. Investigation of protein composition by liquid chromatography–tandem mass spectrometry enabled us to perform an in–depth analysis of the olfactory cleft mucus proteome regarding the diversity of different proteins in individual patients. However untargeted proteomics of the olfactory cleft mucus may not be an applicable approach to develop biomarkers for olfactory disorders. Targeted analyses of distinct proteins known to be involved in olfactory perception but not detected by our approach, e.g. odorant binding proteins, may provide more information regarding pathophysiology of olfactory diseases.

## Introduction

The human proteome is the entire set of proteins expressed by the human genome. A very intriguing possibility of understanding health and disease are proteomic analysis of human fluids, including nasal mucus. To exploit the role of the nasal mucus proteome in the human body we need methods to be able to accurately identify, quantify and annotate proteins in it. Technical advances including liquid chromatography–tandem mass spectrometry (LC–MS/MS) and its data analysis provide us with the opportunity for detailed proteomic analysis of the nasal mucus.

Mucus covers the nasal epithelium. Recent studies revealed that it contains more than 400 proteins with different physiological functions. They have been found to be involved in different molecular processes including anti–oxidative stress, anti–inflammation, protease inhibition, anti–microbial mechanisms and also olfactory perception. Alterations of the nasal mucus proteome may provoke substantial changes of the nasal physiology and have already been associated with rhinologic diseases such as allergic rhinitis^1–6^.

In humans, the olfactory epithelium is located in the olfactory cleft at the upper part of the nasal cavity. In this region, olfactory sensory neurons can be found and olfactory perception takes place. The anatomical barrier to the brain is weakened in this area by sensory axons running through the cribriform plate. The olfactory cleft mucus and epithelium thus have to provide a protective barrier against brain infections. Odorants must transfer through the first barrier built by the mucus in order to be able to reach and stimulate olfactory neurons^7,8^. This process is thought to be facilitated by odorant binding proteins (OBP) IIa and IIb discovered in the human olfactory cleft mucus which may therefore be key players in the sense of smell^2,9,10^. OBPs are supposed to carry lipophilic odorant molecules through the mucosal layer leading to odorant perception^10–13^. Glutathione-S-transferases (GSTs) may be involved in termination of the dynamic process of odorant perception by elimination of odorants^11,12^. Biochemical studies in vertebrate olfactory tissue indicated that certain odorants stimulate adenylyl cyclase in a GTP-dependent manner via GTP-binding protein (G-protein) alpha subunits in vertebrate olfactory epithelium^13^. Débat *et al*., who, to our knowledge, have been the only group investigating the human olfactory cleft proteome so far, reported that the olfactory cleft mucus proteome differed from the mucus proteome of the rest of the nasal cavity, including proteins involved in chemosensory processes^2,9^.

Olfactory disorders are common diseases affecting up to 19% of the general population^14,15^. Dysosmia can be caused by sinunasal and non–sinunasal pathologies. Main sinunasal causes comprise acute or chronic infections of the sinunasal mucosa, inflammatory diseases like chronic rhinosinusitis with or without polyps, allergic rhinitis, as well as anatomic distortions of the nose. Non–sinunasal causes can be a consequence of viral infections of the respiratory organs (postinfectious), chronic meningitis, head trauma, contact with toxic chemicals and neurodegenerative diseases or can be idiopathic without a morphologic correlate^14,16,17^. Despite extensive research, the pathophysiology of postinfectious and idiopathic olfactory loss remains poorly understood. The involvement of either dysfunction of odorant perception at the level of the olfactory neuroepithelium or central olfactory processing pathways is unclear^14,18,19^. To improve the understanding of the pathophysiology of olfactory disorders, a detailed investigation of all levels of olfactory perception is crucial because alterations of the mucosal layer, the first barrier for odorants, may play a role in the etiology of olfactory diseases.

Based on the combined evidence, we felt that further proteomic analyses of the olfactory cleft mucus were warranted. This study was conducted as a pilot study to map the olfactory cleft proteome employing label free quantitation (LFQ), a current technique for proteomic analysis based on quantitation of peak areas of peptide elution profiles summed up resulting in the relative abundance of each protein, and to compare our findings with previously published data^2^. Furthermore, we aimed to investigate proteomic changes as potential biomarkers in patients suffering from idiopathic and postinfectious olfactory disorders compared to healthy controls.

## Materials and Methods

The study was conceived as a pilot study. Participants were recruited from the Department of Otorhinolaryngology, University Hospital of Graz. The study was approved by the Ethics Committee of the Medical University of Graz (1038/2015) and conducted according to the guidelines of the Declaration of Helsinki on biomedical research involving human subjects. All participants provided written informed consent.

### Clinical assessment

Clinical assessment was performed according to the ‘European position paper on rhinosinusitis and nasal polyps’ and the ‘European position paper on olfactory dysfunction’^14,20^. It included clinical interviews, nasal endoscopy, magnet resonance imaging and blood tests (including neurotropic viruses). Olfactory function was assessed using the ‘Sniffin Sticks Test®’ which is a clinically approved test of olfactory function including threshold, discrimination and identification (Burghart, Wedel, Germany)^21^. The olfactory detection threshold was assessed with n–butanol which was presented in 16 dilutions in a staircase, three–alternative, forced–choice procedure. Odor discrimination ability was obtained by presenting 16 triplets of odorant pens. Odor identification was assessed by means of 16 common odors. Possible scores for the detection threshold ranged between one and 16 (with higher scores indexing lower thresholds), and for the other two subtests between zero and 16. The scores for all three subtests were summed to obtain the Threshold–Detection–Identification (TDI) score with a maximum value of 48. The olfactory function level is divided into three categories according to Hummel *et al*.: normosmia (TDI = 48–31), hyposmia (TDI = 30–16), and anosmia (TDI ≤ 15)^22^.

### Study subjects

Subjects between the age of 18 to 65 with diagnosed idiopathic and postinfectious hyposmia and anosmia were included in this study. Subjects with other etiologies of olfactory disorders were excluded. The participants had not taken therapy regarding olfactory dysfunction (including local or systemic glucocorticoids and antibiotics) at least four weeks before their inclusion in the study.

### Sampling procedure

No local medication was applied before sample collection. Nasal mucus was collected from the olfactory cleft of patients after their primary diagnosis of idiopathic or postinfectious olfactory loss as well as in healthy controls under endoscopic control by an otorhinolaryngologist (A.W.). The anterior boundary was the anterior attachment of the middle turbinate, and the posterior boundary was the anterior wall of the sphenoid sinus. The medial boundary was the nasal septum, and the lateral boundary was the middle and superior turbinates^23^. Samples were collected using a special suction device (Sinus Secretion Collector, Medtronic Xomed, Jacksonville, Florida, USA) which was proven to be suitable in previous proteomic investigations of the nose under endoscopic control (rigid endoscopes, 4 mm, 0 degree angled and/or 30 degree angled, Karl Storz, Tuttlingen, Germany)^1,3,5^. Samples were collected taking care not to touch the mucosa in order to avoid damage to the epithelium. Cells such as epithelial cells were eliminated by centrifugation before protein analyses. The sample collection technique provided enough mucus for proteomic analysis from a single collection in all subjects.

500 µl phosphate buffered saline were added to the samples, thoroughly mixed and centrifuged at 12000 g for 5 min to remove insoluble particles. Protein content was estimated by Bradford assay (Bio–Rad, Vienna, Austria). 50 µg of protein was precipitated with four volumes of acetone at −20 °C overnight. The pellet was solubilized in 50 µl 25% 2,2,2-trifluoroethanol (TFE) in 50 mM trishydroxymethylaminomethane–hydrochloride (Tris–HCl) pH = 8.5, reduced with 10 mM tris(2-carboxyethyl)phosphine (TCEP), alkylated with 40 mM chloroacetamide at 95 °C for 10 min and then diluted to 10% TFE with 50 mM ammonium bicarbonate. Protein was digested by adding 1 µg of Promega modified trypsin and shaking overnight at 550 rpm at 37 °C. The resulting peptide solution was acidified by adding 5% formic acid (0.1% end concentration) and stored at −20 °C until further LC–MS/MS analysis.

### LC–MS/MS analysis

LC-MS/MS analysis was performed as previously published^1^ with following changes. An aliquot of 500 ng of each sample was injected in a nano–HPLC (Dionex Ultimate 3000) equipped with a C18, 5 µm, 100 Å, 20 × 0.1 mm, enrichment column and an Acclaim PepMap RSLC nanocolumn (C18, 2 µm, 100 Å, 500 × 0.075 mm) (Thermo Fisher Scientific). Samples were concentrated on the enrichment column for 6 min at a flow rate of 5 µl/min with 0.1% formic acid as isocratic solvent. Separation was carried out on the nanocolumn at a flow rate of 250 nl/min at 60 °C using the following gradient, where solvent A is 0.1% formic acid in water and solvent B is acetonitrile containing 0.1% formic acid: 0–6 min: 4% B; 6–264 min: 4–25% B; 264–274 min: 25–95% B, 274–289 min: 95% B; 289.1–304 min: 4% B; The sample was ionized in the nanospray source equipped with stainless steel emitters (Thermo Fisher Scientific, Vienna, Austria) and analyzed in a Thermo Orbitrap velos pro mass spectrometer in positive ion mode by alternating full scan MS (m/z 300 to 2000, 60000 resolution) and MS/MS by collision-induced dissociation (CID) of the 10 most intense peaks in the ion trap with dynamic exclusion enabled.

### Data analysis

LC-MS/MS analysis was performed as previously published^1^ with following changes. The LC-MS/MS data were analyzed for protein identification and quantification using MaxQuant (version 1.5.8.3) against the human public SwissProt database with taxonomy homo sapiens (downloaded on 02.03.2017) and common contaminants (20233 sequences). Detailed search criteria were used as follows: trypsin, maximum two missed cleavage sites; carbamidomethylation on cysteine as fixed and oxidation on methionine as variable modification; MS/MS ion search with decoy database search included; precursor mass tolerance ±4.5 ppm; product mass tolerance ±0.5 Da; acceptance parameters for identification: 1% peptide to spectrum match (PSM) false discovery rate (FDR); 1% protein FDR; minimum two peptides. In addition, a label free quantitation (LFQ) including the match between runs feature of MaxQuant was performed requiring a minimum of two ratio counts of quantified razor and unique peptides.

Further data processing was performed using Perseus software version 1.5.8.5. Contaminants and reverse protein sequences created during database search were removed. LFQ intensities were log2 transformed in order to lower the effect of outlier values and filtered for five valid values in at least one condition. For protein groups missing LFQ values, this value was obtained by means of an imputation step. Briefly, missing values were replaced with random values taken from a downshifted Gaussian distribution of all values, in order to simulate an LFQ value for those low abundant protein groups. Perseus default parameters (width of 0.3 and downshift of 1.8 separately for each column) were maintained during this step. For statistical analysis, analysis of variance (ANOVA) with subsequent multiple testing correction by permutation–based FDR was used to identify significant protein groups (q ≤ 0.05). The mass spectrometry proteomics data were deposited to the ProteomeXchange Consortium (http://proteomecentral.proteomexchange.org) via the PRIDE partner repository with the dataset identifier PXD009172^24^.

Proteins were assigned to different biological processes and molecular function using Gene Ontology Annotation (https://www.ebi.ac.uk/GOA) and PANTHER (Protein ANalysis Through Evolutionary Relationships, http://www.pantherdb.org,) databases.

### Statistical analysis

Data are reported as means, ± standard errors of the mean (SEM). Statistical analysis by Mann–Whitney–U test, analysis of variances, calculation of Pearson correlation coefficient and multivariate analysis for group differences were performed with SPSS 18.0 software (Chicago, Illinois, USA). A p–value of < 0.05 was considered significant. Power analysis was performed according to the recommendation of Saccenti *et al*. and van Iterson *et al*.^21,25^. Calculation was performed with MetaboAnalyst (http://www.metaboanalyst.ca/) at a power level of 0.8 and a FDR of 5%.

## Results

Seven patients with idiopathic hyposmia and anosmia (seven male, mean age 52.0 ± 5.1 years; TDI = 17.3 ± 2.0), seven patients with postinfectious hyposmia and anosmia (4 male, 3 female; mean age 56.0 ± 3.8 years; TDI = 18.1 ± 3.7) and seven healthy controls (2 male, 5 female; mean age 34.0 ± 4.0 years; TDI = 34.4 ± 0.6) were included in this study. A summary of the clinical parameters of the participants is shown in Tables 1 and ST1.

**Table 1:** Descriptive data of the study population including group comparisons of TDI-scores and protein concentration (IOD = idiopathic olfactory disorder, POD = postinfectious olfactory disorder, C = control, TDI = threshold-discrimination-identification score, vs. = versus, PC = protein concentration, yrs. = years, p = p-value).

Group	Age (yrs.)	Participants		Mean TDI score	In-cohort comparison for TDI		Mean PC [µg/µL]	In-cohort comparison for PC	
		*(n* male)	*(n* female)						
IOD	52.0 ± 5.1	7	0	17.3 ± 2.0	IOD vs. C: p < 0.01	p = 0.7: IOD vs. POD:	0.21 ± 0.03	p = 0.32: IOD vs. C	p = 0.13: I vs. POD
POD	56.0 ± 3.8	3	4	18.1 ± 3.7	POD vs. C: p < 0.01	p = 0.7: POD vs. IOD	0.57 ± 0.19	p = 0.81: POD vs. C	p = 0.13: POD vs. IOD
C	34.0 ± 4.0	2	5	34.3 ± 0.6	C vs. IOD: p < 0.01	p < 0.01: C vs. POD:	0.51 ± 0.31	p = 0.32: C vs. IOD	p = 0.81: C vs. POD
Total	**47**.**7** **±** **3**.**2**	**12**	**9**	**23**.**3** **±** **2**.**0**			**0**.**48** **±** **0**.**06**		

### Profiles of protein composition derived from nasal mucus retrieved from patients and healthy controls

**Figure 1:**
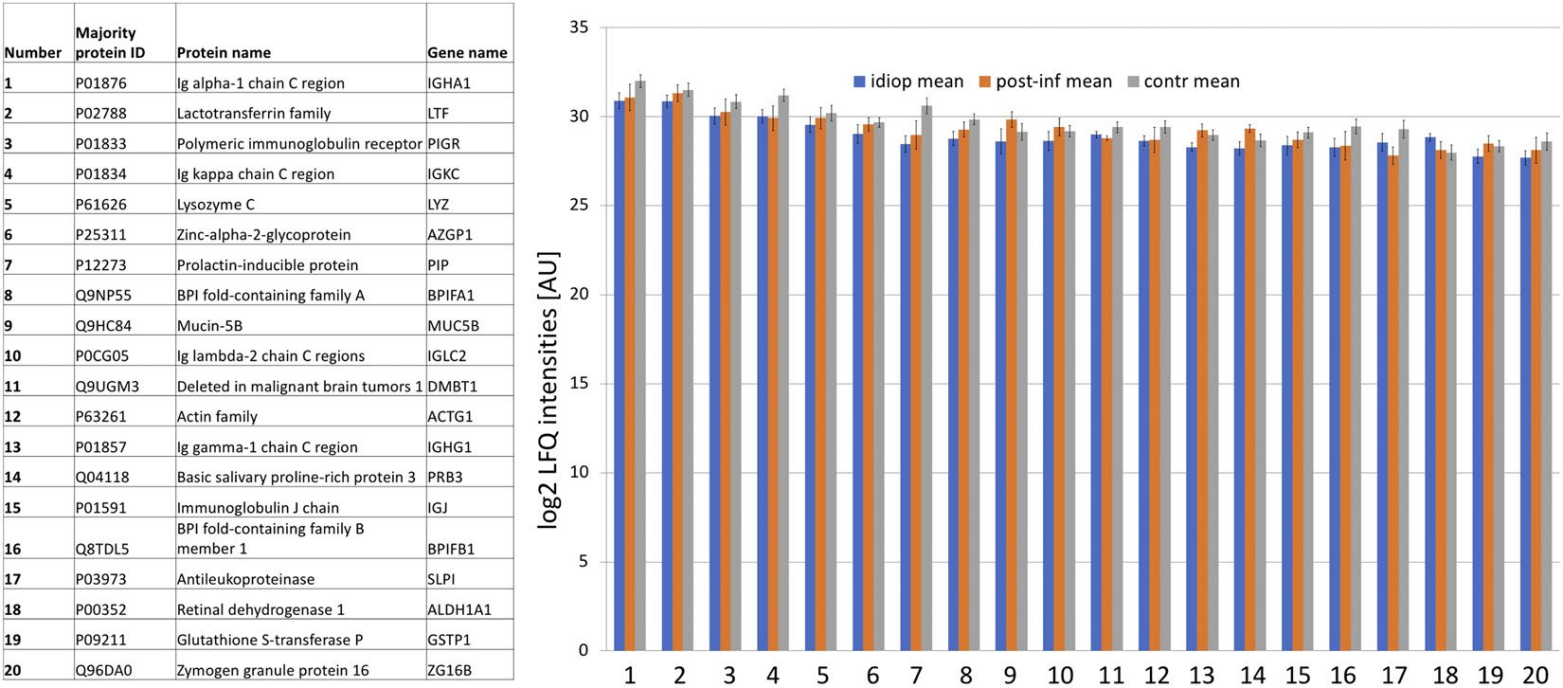
The twenty most abundant proteins in different groups (idiop mean = mean LQF intensity of the idiopathic olfactory disorder group; post-inf mean = mean LQF intensity of the postinfectious olfactory disorder group; contr mean = mean LQF intensity of the control group; error bars = standard error of the mean). No significant differences between different groups were observed. Further details about protein characteristics including assignment to molecular function and biological processes are given in Supplementary Table ST2.

In total, 1117 different proteins were detected after filtering for at least five valid values in at least one group (see Supplementary Table ST2). Overall, the 10 most abundant proteins were: Ig alpha–1 chain C region (IGHA1), Lactotransferrin (LTF), Polymeric immunoglobulin receptor (PIGR), Ig kappa chain C region (IGKC), Lysozyme C (LYZ), Prolactin–inducible protein (PIP), Mucin–5B (MUC5B), BPI fold–containing family A member 1 (BPIFA1) and Zinc–alpha–2–glycoprotein (AZGP1). An overview of the 20 most abundant proteins in different groups is given in Fig. 1.

Using Gene Ontology Annotation, analysis of molecular function and biological processes regarding chemosensory perception and olfaction was performed. Of the identified protein group of the G protein alpha subunit family (P04899, P63096, P08754, P11488, P19087, P09471, A8MTJ3, P38405 and P63092) P38405 was annotated to be involved in sensory perception of smell, and A8MTJ3, P19087 and P11488 in sensory perception of taste next to general signal transduction (see Supplementary Table ST2). Since these 9 G protein alpha subunits are highly homologous they could not be discriminated although seven peptides were sequenced. Two G protein beta subunits (P62879 and P63244) were also identified but have not been implicated in olfaction so far. In addition, we annotated several glutathione-S-transferases (P09211, P30711, Q9Y2Q3, P28161, P78417, and the protein group of P08263, Q16772 and Q7RTV2, see Supplementary Table ST2) involved in cellular oxidant detoxification and glutathione derivative biosynthetic process, some of which may play a role in termination of the olfactory signal by modifying and thereby removing odorants.

**Figure 2:**
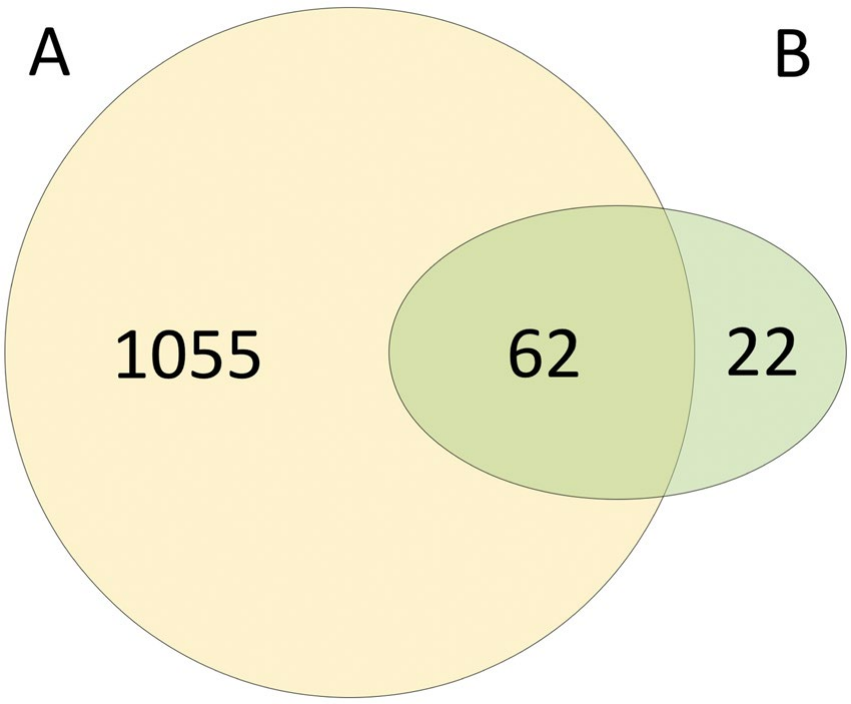
Venn diagram of protein distribution (number of proteins) between results of the present study (**A**) and results of the study by Débat *et al*.^2^ (**B**).

Comparison of results published previously showed that 62 (74%) out of 84 proteins detected by Débat *et al*. were detected in the present study. Odorant binding proteins (Q9NY56, Q9NPH6), were not observed in this investigation while Debàt *et al*. did not report the finding of G proteins and identified only two (P08263, P09211) members of the glutathione-S-transferase family (see Fig. 2)^2^.

**Figure 3:**
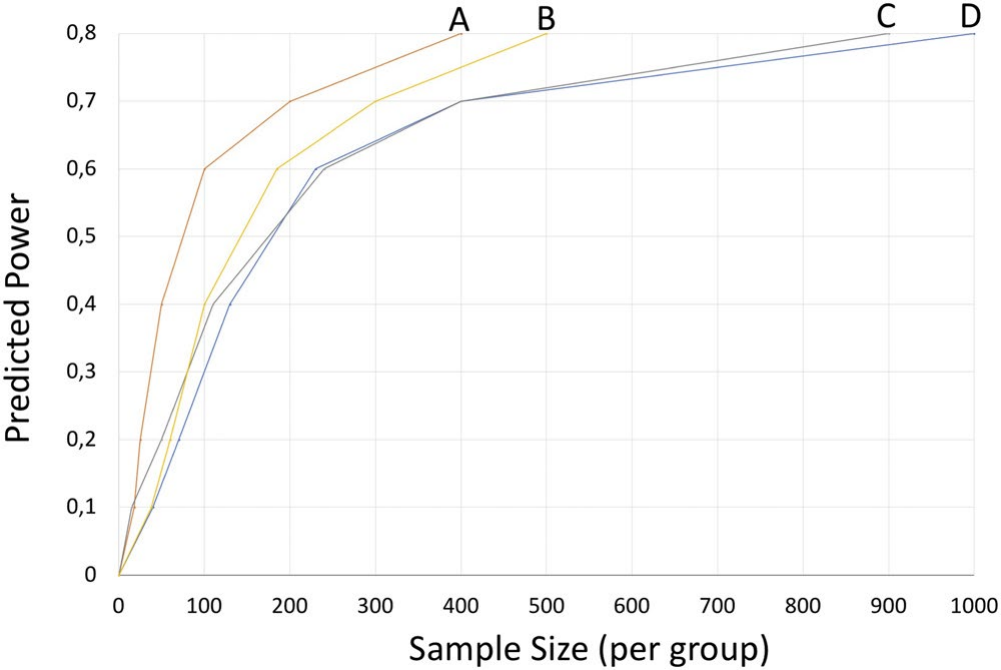
Power analysis for group comparisons based on this pilot study. False discovery rate was set to 5%. (**A)** overall patients vs. control; (**B**) idiopathic olfactory disorder vs. control; (**C**) idiopathic olfactory disorder vs. postinfectious olfactory disorder; (**D**) postinfectious olfactory disorder vs. control.

ANOVA corrected for multi–testing revealed not a single protein out of these 1117 mucus proteins of the olfactory cleft to be significantly altered in patients compared to healthy controls. Based on this data, power-analysis was performed in order to calculate sample sizes for identification of potential biomarkers in the nasal mucus proteome with regard to health and disease. Power analysis based on a power of 0.80 and an FDR of 5% revealed virtual sample sizes for group comparisons as follows: Overall patients vs. control: n = 900; Idiopathic olfactory disorder vs. control: n = 400; Idiopathic olfactory disorder vs. postinfectious olfactory disorder: n = 900; postinfectious olfactory disorder vs. control: n = 500 (see Fig. 3). Furthermore, randomization of all subjects included in this study into two groups revealed a power level of 0.65 in a cohort of 900 subjects (data not shown).

In total, 13 biological processes were enriched in the overall olfactory cleft mucus proteome of the whole study group. The majority of identified proteins were involved in cellular processes (29.7%), metabolic processes (27.1%) and cellular component organization or biogenesis (11.5%) (Tables 2 and ST3).

**Table 2:** Biological processes: Overall number of proteins assigned to different biological processes.

Biological Process	Number of proteins	%
Cellular process	481	30.0%
Metabolic process	431	26.9%
Cellular component organization or biogenesis	185	11.5%
Localization	114	7.1%
Biological regulation	108	6.7%
Response to stimulus	97	6.0%
Multicellular organismal process	56	3.5%
Developmental process	54	3.4%
Immune system process	51	3.2%
Biological adhesion	10	0.6%
Reproduction	8	0.5%
Locomotion	8	0.5%
Growth	1	0.1%
Total	1604	100%

In total, 989 different proteins were assigned to various biological processes. Please note that some proteins were assigned to various processes.

Overall, a total protein concentration of 0.48 ± 0.06 µg/µL was observed, which was not found to be significantly different between groups by Mann Whitney–U–Test (see Table 1 and ST1). Pearson correlation analysis also showed no significant correlation between TDI and protein concentration (r = 0.11, p = 0.62). Furthermore, also age and protein concentration were not correlated (r = 0.06, p = 0.78).

## Discussion

This study aimed to map the olfactory cleft mucus proteome using current techniques for proteomic analysis. Furthermore, we aimed to investigate proteomic changes as potential biomarkers in patients suffering from idiopathic and postinfectious olfactory disorders compared to healthy controls. Despite the high number of identified proteins (N = 1117) in the olfactory cleft proteome, we did not find any evidence that significant differences in the nasal mucus proteome might reflect disease status of patients.

A potential target of proteomic analysis of the olfactory system are odorant binding proteins (OBPs). OBPs have been implicated in the first step of olfactory perception by being involved in the delivery of odorants that have been transported into the nose through the olfactory cleft mucus to the site of olfactory perception. The aqueous solubility of hydrophobic odorants is thought to be enhanced via OBPs which exist in the extracellular fluid surrounding the odorant receptors. The association of different OBP subfamilies with distinct classes of olfactory neurons having different odorant specificities suggests that OBPs can act as selective signal filters, peripheral to the actual receptor proteins^26^. Beside transport of odorant to olfactory receptors, they may also be involved in elimination of odorants^10^. In humans, odorant binding proteins IIa and IIb have been observed on mRNA level^27^. While hOBPIIa is expressed in the oral sphere (nasal mucus, saliva, lachrymal glands), hOBPIIb is expressed in the genital sphere organs. Surprisingly, olfactory binding proteins were absent in the present cohort. Since OBPs are very resistant to organic solvents and proteolytic digestion we may have lost them during our standard proteomic sample preparation although we took care to denature proteins prior to digestion by acetone precipitation and also included trifluoroethanol for solubilisation of hydrophobic proteins prior to digestion^2^. Another explanation for the absence of OBPs in our cohort may be an extremely low concentration of these proteins in the olfactory cleft mucus. Although odorant binding proteins are supposed to play a key role in olfactory perception, their role in humans remains questionable due to the low concentration of proteins in the olfactory cleft mucus in comparison to other mammals^2,10^.

GSTs are supposed to be involved in termination of odorant perception by elimination of odorants. GSTs are hetero- or homo-dimeric proteins of approximately 25 kDa in size^11^. The expression of GSTs as well as their activities have been highlighted in the olfactory organs of several species^12^. Biological functions of these conjugating enzymes include cellular metabolism, oxidative stress response and the detoxification of a wide range of xenobiotic compounds^28^. The biochemical characterization of antennal-restricted GSTs in insects confirmed the activity of such enzymes in odorant conjugation and highlighted the possible contribution of GSTs to the detoxification of compounds that might interfere with odorant detection^29^. This particular localization led to the hypothesis of a possible dual function of GSTs in antennae of insects where they could play a role in signal termination and in odorant clearance, as odorant-degrading enzymes^26^. Although GSTs have been found in the present study we did not observe altered protein abundance in patients compared to healthy controls. Thus, our results did not reveal abundance of GSTs as a potential biomarker for olfactory performance.

Gene Ontology annotation also revealed the presence of G protein family proteins associated with olfaction in the dataset. G proteins are intracellular membrane-associated, heterotrimeric proteins composed of three subunits: alpha (IPR001019), beta (IPR001632) and gamma (IPR001770)^30^. These proteins and their G protein coupled receptors (GPCRs) are highly homologous and have similar functions. Beside sensory perception, they are involved in cell growth and hormonal regulation^31^. Since they are usually localized intracellularly their release into the mucus may be a sign of cellular clearance during turnover/regeneration of the epithelium. On the same note, we also identified cyclase associated actin cytoskeleton regulatory protein 1 (CAP1), which is implicated in the cyclic AMP pathway, a critical node in signal transduction.

To our knowledge, Débat *et al*. have been the only group investigating the human proteome of the nasal mucus directly at olfactory areas so far. They used two approaches: (1) two–dimensional gel electrophoresis followed by tryptic digest and matrix-assisted laser desorption/ionization – time of flight analysis, and (2) reversed phase liquid chromatography of intact proteins followed by Edman sequencing. By combining the results from these two approaches they identified 83 different proteins in 16 healthy adult subjects by pooling 10–12 individuals. Comparison of detected proteins by us and Débat *et al*. reveals considerable differences (see Fig. 2). While Débat *et al.* did not report the presence of G protein family they detected OBPs using Edman sequencing^2^. However, they could not discriminate between hOBPIIa and IIb due to their high homology and also only observed three peptide fragments but failed to detect the intact protein(s) by Western blot. Thus the different proteomic approaches have to be considered in the comparison of results between different studies^2^.

This is the first proteomic study investigating the olfactory cleft mucus in patients with postinfectious and idiopathic olfactory disorders. Viral upper respiratory infection is one of the most commonly identified causes of olfactory loss, comprising about 20 to 30% of patients with olfactory disorders^14,32,33^. In patients where extensive assessment does not reveal a clear underlying aetiology of olfactory dysfunction, it can be classified as idiopathic^14^. Up to 16% of patients suffering from olfactory disorders can be assigned to this group^34^.

Unfortunately, the pathophysiology of postinfectious olfactory loss remains poorly understood. Further studies were recommended in the ‘European Position Paper on Olfactory Dysfunction’^14^ recently, thus we anticipated a proteomic analysis of the olfactory mucus in these distinct groups. However, we failed to identify proteomic biomarker candidates in the olfactory cleft mucus of patients suffering from postinfectious olfactory disorders or idiopathic olfactory disorders compared to healthy controls. Based on our data, a power analysis according to the recommendation of Saccenti *et al*. using MetaboAnalystR for data analysis was performed^21,25^. Approximate sample sizes for group comparisons were calculated to be in the range of 400 to 900 subjects per group. On this basis, it would be virtually impossible to obtain a sufficient number of patients for further analysis and it would also require excessive measurement time in order to reach a power level of 0.8 at a false discovery rate of 5% to find protein biomarkers for these pathologies.

Furthermore, group comparisons of overall total protein concentrations and correlation of protein concentration, TDI and age showed no differences.

The main limitation of this pilot study is its limited sample size and the heterogeneity of the patient and control group in terms of their demographics and clinical parameters. Although no potential driving factors for proteomic abundance were found, these potential confounders of results cannot be excluded.

Overall, the results of this study revealed 1117 different proteins identified in the olfactory cleft mucus in at least five individuals, which is the highest number of proteins identified so far. Although proteins of the G protein family that are associated with olfaction were observed, OBPs were not detected. We also did not find proteomic differences of the olfactory cleft mucous between patients suffering from postinfectious and idiopathic dysosmia and healthy controls. Our findings suggest that investigation of protein composition of the olfactory cleft mucus by untargeted shotgun proteomics is not an applicable method to develop biomarkers for idiopathic and postinfectious olfactory disorders. However, targeted analyses of distinct low abundant proteins (e.g. OBPs) may provide more information regarding the pathophysiology of olfaction diseases on a protein level.

## Electronic supplementary material

Supplementary Table ST1

Supplementary Table ST2

Supplementary Table ST3
